# Eggs modulate the inflammatory response to carbohydrate restricted diets in overweight men

**DOI:** 10.1186/1743-7075-5-6

**Published:** 2008-02-20

**Authors:** Joseph C Ratliff, Gisella Mutungi, Michael J Puglisi, Jeff S Volek, Maria Luz Fernandez

**Affiliations:** 1 Department of Nutritional Sciences, University of Connecticut, Storrs, CT, USA; 2 Department of Kinesiology, University of Connecticut, Storrs, CT, USA

## Abstract

**Background:**

Carbohydrate restricted diets (CRD) consistently lower glucose and insulin levels and improve atherogenic dyslipidemia [decreasing triglycerides and increasing HDL cholesterol (HDL-C)]. We have previously shown that male subjects following a CRD experienced significant increases in HDL-C only if they were consuming a higher intake of cholesterol provided by eggs compared to those individuals who were taking lower concentrations of dietary cholesterol. Here, as a follow up of our previous study, we examined the effects of eggs (a source of both dietary cholesterol and lutein) on adiponectin, a marker of insulin sensitivity, and on inflammatory markers in the context of a CRD.

**Methods:**

Twenty eight overweight men [body mass index (BMI) 26–37 kg/m^2^] aged 40–70 y consumed an ad libitum CRD (% energy from CHO:fat:protein = 17:57:26) for 12 wk. Subjects were matched by age and BMI and randomly assigned to consume eggs (EGG, n = 15) (640 mg additional cholesterol/day provided by eggs) or placebo (SUB, n = 13) (no additional dietary cholesterol). Fasting blood samples were drawn before and after the intervention to assess plasma lipids, insulin, adiponectin and markers of inflammation including C-reactive protein (CRP), tumor necrosis factor-alpha (TNF-α), interleukin-8 (IL-8), monocyte chemoattractant protein-1 (MCP-1), intercellular adhesion molecule-1 (ICAM-1), and vascular cell adhesion molecule-1(VCAM-1).

**Results:**

Body weight, percent total body fat and trunk fat were reduced for all subjects after 12 wk (P < 0.0001). Increases in adiponectin were also observed (P < 0.01). Subjects in the EGG group had a 21% increase in this adipokine compared to a 7% increase in the SUB group (P < 0.05). Plasma CRP was significantly decreased only in the EGG group (P < 0.05). MCP-1 levels were decreased for the SUB group (P < 0.001), but unchanged in the EGG group. VCAM-1, ICAM-1, TNF-α, and IL-8 were not modified by CRD or eggs.

**Conclusion:**

A CRD with daily intake of eggs decreased plasma CRP and increased plasma adiponectin compared to a CRD without eggs. These findings indicate that eggs make a significant contribution to the anti-inflammatory effects of CRD, possibly due to the presence of cholesterol, which increases HDL-C and to the antioxidant lutein which modulates certain inflammatory responses.

## Background

Insulin resistance is recognized as the major defect leading to the development of the metabolic syndrome including a proinflammatory state. Experimental and epidemiological evidence reveals that chronic inflammation is an independent predictor of cardiovascular disease (CVD) [1] and is directly involved in the promotion of insulin resistance and atherosclerosis [2-4]. Infections or the healing of wounds produce an inflammatory response which attracts leukocytes to a localized area of the vasculature and permits passage through the underlying tissue [5]. Repeated stimulation of this inflammatory response can lead to the development of several chronic diseases mediated by increased levels of cytokines and chemokines. Adipose tissue secretes several adipokines, such as tumor necrosis factor-alpha (TNF-α), interleukin-8 (IL-8), and adiponectin which can modulate lipid and glucose metabolism [6]. Adiponectin is a unique hormone that is both anti-inflammatory and anti-atherogenic and low circulating levels are found in obese, insulin resistant individuals. Elevated levels of the acute-phase reactant C-reactive protein (CRP) and TNF-α are associated with an increased risk for numerous chronic diseases [7]. Monocyte chemoattractant protein-1 (MCP-1) is a proinflammatory chemokine produced in response to inflammatory stimuli like TNF-α. Additionally, upregulation of vascular cell adhesion molecule-1 (VCAM-1) and intercellular adhesion molecule-1 (ICAM-1) signifies the initiation of atherogenesis by permitting circulating cells to adhere to the endothelium [8-10].

Acute carbohydrate consumption has been shown to stimulate reactive oxidative species while initiating many pro-inflammatory processes [11]. Additionally, isocaloric high carbohydrate diets are associated with several markers of inflammation [12]. Carbohydrate restricted diets (CRD) are able to reduce biomarkers of inflammation in the absence of weight loss [13,14]. Dietary carbohydrate, rather than fat, plays a critical role in activating pro-inflammatory processes through their effect on the fatty acid composition of lipids and membranes [15]. Endogenous fatty acids function as ligands for receptors and transcription factors that modulate inflammatory signaling cascades. CRDs are ideal for reducing inflammation because they reduce plasma glucose excursions, the major stimulus for pancreatic secretion of insulin, and modulate the underlying factors associated with CVD and the metabolic syndrome.

Eggs are a breakfast staple that are affordable and provide a good source of protein and other valuable nutrients. Experimental studies show that the additional dietary cholesterol provided by whole eggs does not increase the risk of CVD in a variety of sample populations [16,17]. Additionally, whole eggs contain the potent antioxidant, lutein, which protects against numerous inflammatory processes [18]. To our knowledge this is the first study measuring inflammatory marker responses to a CRD utilizing eggs as a source of dietary cholesterol and lutein.

We have previously shown that during an intervention following a CRD, adult men consuming eggs presented a significant increase in plasma HDL-C (P < 0.0001) compared to those individuals who were consuming egg substitute [19]. The purpose of this study was to determine whether the observed increases in HDL-C [19], plus the fact that eggs are a good source of bioavailable lutein [17], would affect the inflammatory response. Our initial hypothesis was that the additional dietary cholesterol provided by eggs would not modify the beneficial effects of the CRD.

## Methods

### Materials

Liquid whole eggs and cholesterol/fat-free eggs (placebo) were purchased from the Vistar Corporation (Windsor, CT). Aprotinin, sodium azide and phenylmethylsulfonyl fluoride were obtained from Sigma Chemical (St. Louis, MO). Human Cytokine, Human CVD Panel 1, and Human CVD Panel 2 Lincoplex kits were obtained from Linco (Linco Research, Inc, St.Charles, MI).

### Subjects

Thirty-one healthy men aged 40–70 y, with a BMI between 25 and 37 kg/m^2 ^were recruited from the university and the surrounding community[19]. All subjects gave a written informed consent to participate and the Committee on the Use of Human Subjects in Research of the University of Connecticut approved the study protocol.

### Study Design

The CRD was similar to those implemented in our previous studies[14], we conducted this study to determine the effects of egg intake in conjunction with a CRD on the variables for the classification of the metabolic syndrome [19] and on body composition and inflammation markers presented in this manuscript. Briefly, subjects (n = 28) were matched by age and BMI (body mass index) and then randomly assigned, single blinded, to eggs (EGG, n = 15) (640 mg cholesterol) or placebo (SUB, n = 13) (no added cholesterol) for twelve weeks. Both products were equal in consistency and color. All subjects attended a group meeting before the intervention with registered dieticians where detailed instructions on keeping diet records and following a CRD similar to those used in our previous studies [14,20] were discussed. This was a free living food study and no food was provided to subjects. The diet was designed to restrict carbohydrates (~10% of total calories) so that ketones are produced. Subjects were also instructed to abstain from consuming eggs (other than those provided by the study) during the whole intervention. To monitor compliance to the ketogenic diet, subjects used Ketostix reagent strips to assess ketonuria nightly and returned their empty egg containers weekly. Subjects were asked to maintain their habitual level of exercise throughout the intervention. One week prior to the start of the study, subjects completed a 3 day food record to assess habitual food intake. Blood samples, DEXA, food records and anthropometrics were collected at baseline and week 12.

### Blood collection

After an overnight fast, blood was collected into EDTA from an antecubital vein. Plasma was separated by centrifugation at 2000 × g; 20 min, and aprotinin (0.5 mL/100 mL), sodium azide (0.1 mL/100 mL), and phenylmethylsulfonyl fluoride (0.1 mL/100 mL) were added for preservation. Plasma was stored in individual aliquots at -80°C for analysis of cytokines.

### Plasma lipids, lutein, and insulin

Plasma triglycerides and total cholesterol were measured by enzymatic analysis and HDL-cholesterol by measuring cholesterol in the supernatant following precipitation of the apo B containing lipoproteins as previously reported [16]. LDL-C was estimated by the Friedwald equation [21]. Plasma lutein was measured using Waters' HPLC system [22] and detection was measured at 450 nm. Insulin was measured with multiplex assay kit based on the Luminex × MAP technology (Linco Research, Inc, St. Charles, MI).

### Body Weight and DEXA

Weight was measured to the closest 0.25 kg and height to the closest 1 cm on a portable stadiometer/scale. Body mass and body composition were measured in the morning after an overnight fast. Body mass was recorded to the nearest 100 g on a calibrated digital scale with subjects wearing only underwear. Whole body and regional body composition was assessed using a state-of-the-art fan-beam dual-energy X-ray absorptiometry (Prodigy™, Lunar Corporation, Madison, WI). Analyses were performed by the same blinded technician.

### Fasting Adiponectin, sVCAM-1 and sICAM-1

From fasting plasma, VCAM-1, ICAM-1, and adiponectin were measured in duplicate in the same assay using the Human CVD Panel 1 Lincoplex kit. Samples were diluted 1:100 and simultaneously quantified by using Antibody-Immobilized beads and Luminex × MAP technology. All assays were carried out in the same day to decrease variability. The CV was between 2–6%. The sensitivity for VCAM-1, ICAM-1, and adiponectin were 16.0 pg/ml, 9.0 pg/ml, and 56.0 pg/ml, respectively.

### Fasting TNF-α, IL-8, and MCP-1

Plasma TNF-α, IL-8, and MCP-1 concentrations were measured in duplicate in the same assay from a fasting sample using the Human Cytokine Lincoplex kit, which is a multiplex assay kit based on the Luminex × MAP technology (Linco Research, Inc, St. Charles, MI)[23]. This method uses Antibody-Immobilized beads for simultaneous quantification of TNF-α, IL-8, and MCP-1. All assays were carried out in the same day to decrease variability. The CV was between 3–6%. The sensitivity for this assay was 0.66 pg/ml, 1.12 pg/ml, and 1.29 pg/ml, respectively.

### Fasting CRP

CRP concentration was measured in duplicate from fasting plasma with a 1:2000 dilution using the Human CVD Panel 2 (acute-phase proteins) Lincoplex kit. Antibody-Immobilized beads were analyzed using Luminex × MAP technology. The sensitivity of this assay was 6.0 pg/ml.

### Statistical Analysis

Data are presented as means ± SD. A two-way repeated measures ANOVA was used to determine the effects on each subject on all parameters. Each individual's response to the intervention over time was considered as the repeated measure and egg vs. egg substitute the between subject factors. Pearson correlations were used when appropriate and differences of P < 0.05 were considered significant.

## Results

### Diet and Plasma Lipids

In spite of the ad libitum dietary intervention, caloric intake decreased significantly for all subjects (n = 28) from 10243 ± 4039 kJ at baseline to 7967.82 ± 2401 kJ at week 12 (P < 0.05). There were significant changes in macronutrient intake during the intervention. Carbohydrate intake decreased from 42% of total calories at baseline to 17% at week 12 (P < 0.001). Protein intake increased from 17.8% at baseline to 25.8% at week 12 (P < 0.001). Fat intake increased from 39.6% of total energy at baseline to 55.6% at week 12 (P < 0.001). These changes were consistent among the 28 subjects irrespective of the dietary group. However, dietary cholesterol increased from 319 ± 150 g/d at baseline to 827 ± 192 g/d at week 12 (P < 0.001) for the EGG group while there were no changes in dietary cholesterol for the SUB group. Values were 354 ± 170 g/d at baseline and 277 ± 100 g/d after 12 wk (P > 0.15). A more detailed description of the diet has been previously reported [19]

**Figure 1:**
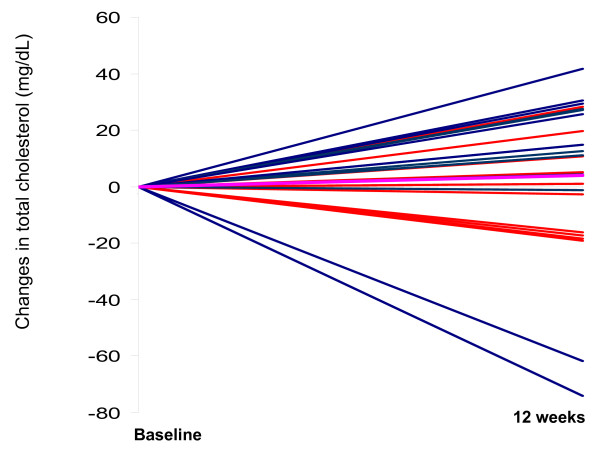
**Changes in total cholesterol between baseline and post-treatment for subjects from the EGG group (blue lines, n = 15) and the SUB group (red lines, n = 13).** The pink line represents the mean for the group.

There was a significant increase in HDL-C for subjects in the EGG group after 12 wk of the intervention (P < 0.001) from 47.6 ± 15,1 mg/dL to 57.1 ± 15.1 mg/dL while the subjects in the SUB group did not change their HDL-C (50.0 ± 9.1 mg/dL at baseline and 48.9 ± 8.8 mg/dL post-intervention). Further, 13 out of 15 subjects had an increase in HDL-C in the EGG group while only 3 out of 13 subjects had an increase in the SUB group [19]. Plasma triglycerides were decreased for all subjects by an average of 45% (P < 0.0001). The combined values for triglycerides were 120.2 ± 59.4 mg/dl at baseline and 73.4 ± 26.9 mg/dl after week 12. There were no changes in total cholesterol observed in either group (193.3 ± 37.9 mg/dl at baseline and 194.8 ± 40.7 mg/dl at week 12). Individual responses for total cholesterol between baseline and 12 weeks are presented in Figure 1 for both the EGG and the SUB groups. There was a great variation in the response to diet going from + 42 mg/dL to -74 mg/dl changes in total cholesterol between baseline and 12 wk. There were no significant changes in LDL-C between baseline and post-treatment (P > 0.05) for either group. Values were 117.8 ± 37.8 mg/dl at baseline and 132.9 ± 43.6 mg/dl after week 12.

### Plasma and Dietary Lutein

Plasma lutein was only increased in the EGG group (P < 0.01) from 0.54 ± 0.24 μmol/L to 0.93 ± 0.42 μmol/L while there were no changes in plasma lutein for the SUB group (0.53 ± 0.29 μmol/L at baseline to 0.53 ± 0.35 μmol/L after 12 wk)

### Anthropometrics and Insulin

**Figure 2:**
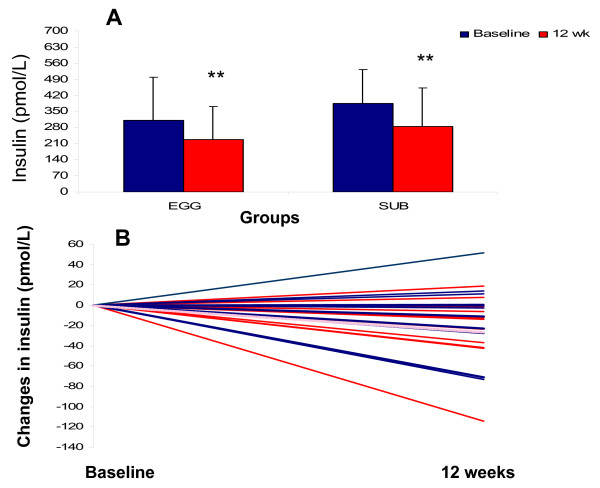
**Concentrations of insulin for subjects from the EGG (n = 15) or the SUB (n = 13) groups at baseline and at week 12.** ** indicates significantly different from baseline (Panel A). Individual responses to insulin are indicated in Panel B for subjects from the EGG group (blue lines, n = 15) and the SUB group (red lines, n = 13). The pink line represents the mean for the group

Body weight and fat mass were decreased significantly for both the EGG and SUB group after the intervention (P < 0.0001) (Table 1). Body weight decreased 6.7 kg and 5.9 kg for the EGG and SUB groups, respectively. Likewise percent body fat and percent trunk fat were significantly decreased for both groups after 12 wk (P < 0.0001). Changes in plasma insulin between baseline and 12 wk for the EGG and SUB groups are presented in Figure 2, **panel A**. Individual changes for all subjects are presented in Figure 2, **Panel B**. Changes in insulin went from an increase of 51 pmol/L to a decrease of -114 pmol/L with a mean change of -26 pmol/L.

**Table 1:** Body weight and body composition as measured by DEXA at Baseline and following 6 and 12 weeks of a carbohydrate restricted diet with 3 eggs per day (EGG group) or the equivalent of egg substitute (SUB)^1^

Variable	Body Weight (Kg)	Total Body Fat (Kg)	Trunk Fat (Kg)
**EGG**			
Baseline	98.9 ± 15.3	30.2 ± 8.0	18.6 ± 5.5
12 Weeks	92.2 ± 12.7	25.4 ± 8.4	15.3 ± 5.7
**SUB**			
Baseline	97.6 ± 19.9	31.6 ± 12.4	19.8 ± 7.8
12 Weeks	91.7 ± 15.7	27.0 ± 12.0	16.6 ± 7.4
Time Effect	P < 0.0001	P < 0.0001	P < 0.0001
Diet Effect	NS	NS	NS
Interaction	NS	NS	NS

^1^Values are means ± standard deviation. EGG n = 15, SUB n = 13. Data were analyzed using repeated measures ANOVA.

### Adiponectin, CRP, VCAM-1, and ICAM-1

**Figure 3:**
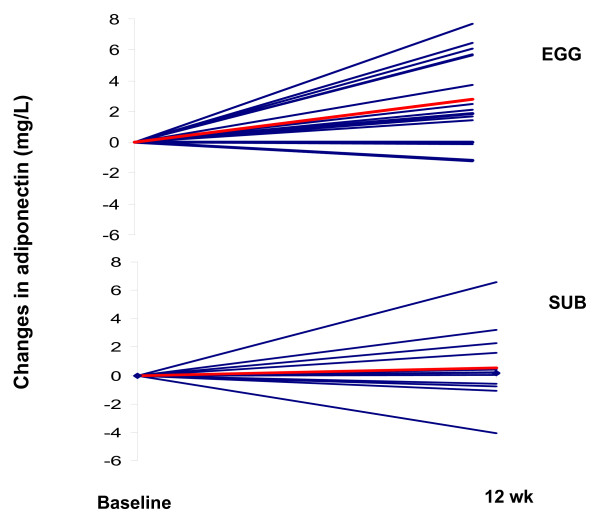
Changes in adiponectin between baseline and post treatment for subjects from the EGG (panel A, n = 15) or the SUB (panel B, n = 13) group. The red line represents the mean for the group.

The fasting values of adiponectin, CRP, VCAM-1, ICAM-1, are presented in Table 2. Adiponectin was significantly increased (P < 0.01) for both groups, with a higher increase in the EGG (15.3 g/L to 18.5 g/L) (P < 0.05). The individual changes in adiponectin for all subjects (n = 28) are presented in Figure 3. There was an increase in adiponectin in 12 subjects from the EGG group from a total of 15 while only 7 out of 13 subjects from the SUB group presented an increase in adiponectin (Figure 3). CRP was only decreased in the EGG group (P < 0.05) Values were reduced from 5.95 mg/L to 4.33 mg/L while there was a non-significant increase in the SUB group (Table 2). There were no differences in ICAM-1 or VCAM-1 observed in either group.

**Table 2:** Fasting serum Adiponectin, CRP, VCAM-1, ICAM-1 at Baseline and following 12 weeks of a carbohydrate restricted diet with 3 eggs per day (EGG group) or egg substitute (SUB).

Variable	Adiponectin (mg/L)	CRP (mg/L)	VCAM-1 (mg/L)	ICAM-1 (mg/L)
**EGG**				
Baseline	15.8 ± 4.6^a^	5.9 ± 2.9^b^	1.13 ± 0.15	0.21 ± 0.05
12 Weeks	18.6 ± 3.8^b^	4.3 ± 3.7^a^	1.19 ± 0.13	0.21 ± 0.04
**SUB**				
Baseline	15.8 ± 5.9^a^	8.6 ± 6.5^bc^	1.17 ± 0.26	0.23 ± 0.06
12 Weeks	16.4 ± 5.2^a^	12.2 ± 11.5^c^	1.14 ± 0.30	0.21 ± 0.07
Time Effect	P < 0.01	NS	NS	NS
Diet Effect	NS	NS	NS	NS
Interaction	P < 0.05	P < 0.05	NS	NS

Values are means ± standard deviation. EGG n = 15, SUB n = 13. Data were analyzed using repeated measures ANOVA. Values in the same column with different superscript indicate significantly different at P < 0.05.

### TNF-α, IL-8, and MCP-1

The fasting values of TNF-α, IL-8, and MCP-1 before and after the intervention are presented in Table 3. MCP-1 was significantly reduced (P < 0.05) for the SUB group after the intervention, while no change was observed in the EGG group (interaction effect, P < 0.05). There were no changes in TNF-α or IL-8 observed in either group.

**Table 3:** Fasting serum TNF-α, IL-8 and MCP-1 concentrations at Baseline and following 6 and 12 weeks of a carbohydrate restricted diet with 3 eggs per day (EGG group) or placebo (SUB).

Variable	TNF-α (ng/L)	IL-8 (pg/L)	MCP-1 (μg/L)
**EGG**			
Baseline	1.5 ± 0.9	1.3 ± 0.7	199.0 ± 87.1^a^
12 Weeks	1.9 ± 0.9	1.3 ± 0.7	194.5 ± 111.7^a^
**SUB**			
Baseline	2.0 ± 0.9	1.7 ± 0.8	224.2 ± 115.8^a^
12 Weeks	2.0 ± 0.9	1.6 ± 0.7	157.0 ± 72.0^b^
Time Effect	NS	NS	P < 0.05
Diet Effect	NS	NS	NS
Interaction	NS	NS	P < 0.05

Values are means ± standard deviation. EGG n = 15, SUB n = 13. Data were analyzed using repeated measures ANOVA. Values in the same column with different superscripts are significantly different at P < 0.05.

## Discussion

In this study we are reporting for the first time that eggs modulate the response of certain inflammatory cytokines during a weight loss intervention using a CRD. Subjects consuming the eggs presented a better response to adiponectin and CRP, two major markers of inflammation and of CHD risk. This effect could be due to the high concentration of lutein, a potent antioxidant present in the egg yolk.

### Adiponectin

There was a significant increase in adiponectin in both the EGG and SUB groups, with a greater increase in adiponectin observed in the EGG group. An anti-atherogenic adipokine, adiponectin decreases adhesion molecule expression that occurs after inflammation [24] and also decreases TNF-α production by macrophages [25]. Reductions in weight and fat mass in obese individuals is often accompanied by an increase in serum adiponectin [26], accordingly, we observed a significant reduction in trunk fat in this study following the intervention. Inflammatory cytokines suppress adiponectin expression [27], so the increase in adiponectin may also be directly related to reduction in these inflammatory markers and because of a selective decrease of CRP only by eggs, a higher increase of adiponectin was observed for this group. Paraoxonase is an antioxidant carried on HDL that protects against LDL oxidation [28], and with inflammation, there is a reduction in paroxonase levels [29]. The increased HDL-C and additional adiponectin observed in the EGG group may have resulted in elevations of paraoxonase, which may explain improvements in the inflammatory profile. Direct administration of adiponectin stimulates fatty acid oxidation in hepatic and muscle tissues, which leads to decreased triglycerides [30]. Increased adiponectin levels are associated with increased insulin sensitivity, decreased triglycerides, and increased HDL-C [31]. Accordingly, we found that the EGG group presented a significant increase in plasma HDL-C in addition to higher levels of adiponectin. Egg intake is associated with significant increases in HDL-C and concentration of anti-atherogenic large HDL particles[16,17]. Large HDL particles are correlated with decreased CHD risk [17,32] because they can more efficiently remove excess cholesterol by returning it to the liver for excretion. It is postulated that adiponectin may influence reverse cholesterol transport (RCT) by increasing HDL production in the liver by promoting apoA-1 synthesis and the ABCA1 pathway [33]. Additionally, accelerated RCT results in decreased secretion of ApoB-100 containing lipoproteins [34], which is evidenced by the reduction in triglycerides. Lipoprotein lipase (LPL) catalyzes the hydrolysis of triglycerides from ApoB-100 containing particles. Several studies show that adiponectin is highly correlated with LPL activity [35,36]. In addition, there were significant reductions in insulin for both groups. Carbohydrate restriction improves insulin resistance by lowering insulin levels and the disinhibition of hormone sensitive lipase, promoting triacylglycerol hydrolysis [13]. As a result, there is an increase in cellular fatty acid uptake and oxidation [13]. The increase in fatty acid oxidation decreases hepatic triglyceride production and the synthesis and secretion of triglyceride-rich VLDL particles. Taken together, these data suggest that interventions which increase HDL may also be directly involved in improving the inflammatory response and increasing insulin sensitivity as demonstrated by the increased levels of plasma adiponectin.

### CRP

At week 12, CRP was reduced only in the EGG group. Serum CRP is an important marker of vascular inflammation and can be used to predict atherosclerosis [7]. Reduced CRP levels are found after weight loss interventions and are inversely correlated to adiponectin [37]. The reduction in adipose tissue due to weight loss reduces the number of pro-inflammatory cytokines (TNF-α, IL-8.) secreted by monocytes, and the number of macrophages and endothelial cells present in the adipocytes [7]. Macrophages and endothelial cells produce CRP, so the reductions in adipose tissue as well as a reduction of carbohydrates work in parallel to mediate the reduction in CRP. Previously, Tannock et al. [38], found that the addition of 4 whole eggs/day for 4 weeks was associated with increased levels of CRP in lean, insulin sensitive individuals, however, there was no effect on CRP in obese or insulin resistant individuals. The subjects in the current study were obese individuals undergoing weight loss and we found that additional dietary cholesterol lowered CRP levels. In previous studies, diets high in monounsaturated fatty acids and polyunsaturated fatty acids have been associated with decreased serum CRP [39]. Additionally, eggs contain the potent antioxidants lutein and xeazanthin, which might play a role in the reduction of inflammation observed in the EGG group. Both groups had significant increases in dietary intake of lutein, but only the EGG group experienced an increase in plasma. These findings correspond with results from another study that found increases in the size and number of HDL particles is associated with increased plasma lutein [18]. We speculate that the reduction of CRP in the EGG group may be the result of increased HDL-C preventing CRP induced upregulation of inflammatory adhesion molecules, possibly through enhanced paraoxonase activity from antioxidants and fatty acids contained within the egg yolk, which can break down oxidized lipids to counteract proinflammatory effects.

### TNF-α, IL-8, and MCP-1

TNF-α is an inflammatory cytokine that is secreted 7.5 times higher from the abdominal adipose tissue of obese subjects than their lean counterparts [40]. A few experimental studies show that weight loss through short-term caloric restriction can reduce circulating levels of TNF-α [14,41], however a reduction of fat mass does not always signal a reduction in TNF-α [42,43]. We did not observe a change in fasting TNF-α in either experimental group. These discrepancies are likely the result of the duration and method of weight loss. Further studies are needed to understand the relationship between TNF-α and central obesity. Additionally, IL-8 did not change after the intervention either. Stimulated by oxidized low-density lipoprotein, IL-8's released from macrophages plays a key role in the development of atherosclerosis [44]. IL-8 allows adhesion of monocytes to the endothelium [45], which promotes the atherosclerotic process. TNF-α stimulates IL-8 synthesis in the adipocytes [46], so the equivocal results found for IL-8 production likely relates to the failure of TNF-α levels to change after the intervention.

MCP-1 levels were significantly reduced for the SUB group at week 12, while no change was observed for the EGG group. MCP-1 is an inflammatory chemokine mostly produced by endothelial cells and macrophages. In visceral adipose tissue, an elevation in MCP-1 is associated with an increased ability to attract macrophages, which reinforces the inflammatory cycle [25]. In endothelial cells, leptin secreted from adipose tissue enhances MCP-1 synthesis [47]. At this point, it is not clear why the SUB group had a greater reduction in MCP-1 compared to the EGG group. However, the reduction of MCP-1 observed in both groups, although not significant in the EGG group, may be the result of decreased circulating leptin due to weight loss in the current study. We have also observed that subjects in both groups reduced levels of leptin following the CRD intervention (Ratliff and Fernandez, unpublished observations).

### VCAM-1 and ICAM-1

VCAM-1 and ICAM-1 levels did not change throughout the intervention for either group. Circulating adhesion molecules play an active role in the atherogenic process by adhering circulating leukocytes into vessel walls. Proteolysis of membrane bound molecules releases soluble forms of VCAM-1 and ICAM-1 into the bloodstream, which can serve as markers of endothelial inflammation [48,49]. Endothelial VCAM-1 and ICAM-1 are upregulated in response to TNF-α [50]. In the current study, there was no change in TNF-α observed in either group, which may result in the unchanged serum VCAM-1 and ICAM-1 values. A previous study found that caloric restriction in conjunction with exercise for one year lowered plasma VCAM-1 and ICAM-1 in overweight women [51]. The brief duration of the current study, gender, or the lack of structured exercise might also explain why there was not a reduction in cellular adhesion molecules for either group.

## Conclusion

We conclude that daily intake of 3 eggs while following a CRD results in a significant decrease in CRP and a more pronounced increase in adiponectin, thus, improving the inflammatory profile. These findings suggest a role of eggs due to their cholesterol and antioxidant content in achieving maximum benefits with carbohydrate restricted diets on certain inflammatory cytokines.

## Abbreviations

BMI, body mass index; CHD, coronary heart disease; CHO, carbohydrate; CRD, carbohydrate restricted diet; CRP, C-reactive protein; CVD, cardiovascular disease; EGG, egg group; ICAM-1, intercellular adhesion molecule;IL-8, interleukin-8; LPL, lipoprotein lipase MCP-1, monocyte chemoattractant protein-1; RCP: reverse cholesterol transport; ROS, reactive oxidative species; SUB, substitute group TNF-α, tumor necrosis factor; sVCAM-1, vascular cell adhesion molecule.

## Competing interests

Supported by a grant from the American Egg Board-Egg Nutrition Center to MLF.

## Authors' contributions

JR was responsible for data acquisition, analysis, and interpretation and for drafting of the manuscript. GM was responsible for data acquisition, analysis and interpretation and critical revision of the manuscript. MP was responsible for data analysis and critical revision of the manuscript. JSV was responsible for study conception and design, data collection, analysis and interpretation and for critical revision of the manuscript. MLF was responsible for study conception and design, data collection, analysis and interpretation, and for critical revision of the manuscript. All authors read and approved the final manuscript.
